# Care Their Diet and Mind: Association between Eating Habits and Mental Health in Chinese Left-behind Children

**DOI:** 10.3390/nu14030524

**Published:** 2022-01-25

**Authors:** Kaixin Liang, Sitong Chen, Xinli Chi

**Affiliations:** 1 Center for Mental Health, School of Psychology, Shenzhen University, Shenzhen 518060, China; liangkaixin2020@email.szu.edu.cn; 2 Institute for Health and Sport, Victoria University, Melbourne 8001, Australia; sitong.chen@live.vu.edu.au

**Keywords:** eating habits, mental health, adolescents, left-behind children, breakfast, fruit intake

## Abstract

Studies have shown that the prevalence of mental health problems is high among left-behind children (LBC). Modifiable eating habits may play an important role in promoting mental health among these adolescents. This study aimed to investigate the situation of dietary habits and their association with mental health problems in a sample of Chinese LBC. We conducted this cross-sectional study in an economically disadvantaged area of China in May 2020. Information on socio-demographic factors, eating habits (the frequency of eating carbohydrates, fruits, vegetables, protein, and breakfast), and mental health problems (insomnia, depression, and anxiety) were collected among local adolescents. Among 8939 adolescents were recruited, a total of 3314 LBC (1455 males and 1859 females, aged 12–17 years) were identified and included in the current study. Associations between dietary habits and mental health were analyzed by generalized linear models. Results indicated that the dietary pattern was unhealthy among LBC and even worse in females. Compared to routinely eating breakfast and fruits, lower frequencies of eating breakfast and fruits were identified as two significant factors with higher risks of mental health problems. Moreover, the association was more pronounced in females. Therefore, improving diet quality, particularly the frequency of eating breakfast and fruit intake may be an effective approach in the mental health promotion for LBC, especially for females.

## 1. Introduction

“Left-behind children (LBC)” in China is a serious social issue that has aroused much attention in recent years. LBC refers to children under 18 years old who are left behind in the registered residence (usually the birthplace) when one or both parents work elsewhere [1]. LBC are more common in socio-economically deprived provinces than in relatively socio-economically developed provinces. LBC are usually taken care of by their grandparents or other relatives from the extended family. As a result, LBC often grow up in families with poor economic conditions, and the care and support from parents is insufficient. The occurrence of mental health problems in LBC in China is a prevalent phenomenon [2], on which relevant researchers and practitioners have shed light and provided reliable evidence. Evidence has demonstrated that the incidence of severe mental health status in LBC was approximately 2.7 times greater than that in non-LBC [3]. A systematic review and meta-analysis reported that the prevalence of depression among LBC was 26.4% [4]. It was reported that the prevalence of mental health problems among LBC in Sichuan province was as high as 43.4% [5]. These data indicate a high prevalence of mental health problems in LBC. Due to the prevalent mental health problem in LBC, it is necessary to pay more attention to the mental health of LBC and put forward effective improvement schemes. The initial step is to identify the factor influencing the mental health status of LBC.

Accumulating evidence has demonstrated that eating habits play a prominent role in mental health promotion or mental disorder prevention in adolescents. Evidence from a synthesized review confirmed that diet can be used as a strategy for the prevention and treatment of mental illnesses among adolescents [6]. Some single empirical studies also supported this view. For instance, the frequency of carbonated soft drinks was associated with a higher frequency of loneliness and anxiety in adolescents [7]. Conversely, there was also an inverse association between fruit and vegetable consumption and mental health problems in adolescents [8]. A pooled analysis using data from 25 low- and middle-income countries indicated that fruit and vegetable intake was related to a lower risk of mental health problems in adolescents [9]. In addition to the specific food intake, breakfast consumption is also considered an integral part of healthy eating habits. Indeed, a meta-analysis concluded that skipping breakfast was positively associated with odds of depression [10]. These studies point to the favorable roles of optimal eating habits on mental health in young people.

However, to our knowledge, there is no evidence concerning the association between eating habits and mental health problems in Chinese LBC. Exploring the relationship between dietary habits and mental health would be of great significance to promote the mental health of LBC, as one’s eating habits are modifiable and daily behaviors. In this regard, if researchers can determine the association between eating habits and mental health, improving diet quality would be a promising way for improving LBC’s mental health. Therefore, this study aimed to explore the association between eating habits and mental health problems (e.g., depression, anxiety, insomnia) among Chinese LBC. Given the existing evidence of an association between diet habits and youth mental health, we expected to observe a significant association between eating habits and mental health problems in LBC.

## 2. Methods

### 2.1. Study Design and Participants

We conducted this cross-sectional study in Hechi city, one of the poorest cities in Guangxi Province, China. Guangxi Province is an economically undeveloped province in China, and there are a large number of LBC [11]. To address the research gap related to the mental health of LBC, Guangxi Province is a suitable testing ground for performing this study. This study was conducted in May 2020, when China had entered the fifth stage (ongoing prevention and control) of fighting the pandemic [12]. The pandemic situation has been generally under control in the mainland, China, presenting a positive momentum, and nationwide virus control was being conducted on an ongoing basis. Vigorous efforts to resume work and reopen schools have been made in China, including Guangxi Province. With the collaboration of the Yizhou District Education Bureau, we recruited 13 local middle schools to participate in our survey. With the assistance of participating schools, students completed our questionnaire via the Wenjuanxing system (a commonly used online survey platform in China, https://www.wjx.cn/, accessed on 20 December 2021). Before filling the questionnaire, participants needed to read through the questionnaire instruction with the option of informed consent at the end. After clicking the option “I agree to participate in the survey”, consented participants could access the questionnaire. As the system allowed voluntary pause or withdrawal at all lengths of the questionnaire, participants could exit the system whenever they wished to do so.

After the survey, we received 8939 submissions. Among them, 3332 students reported “yes” to the question “whether one or both of your parents had migrated to another city for employment and had been separated from your migrant parents for a long time in the past year?” After the exclusion of 18 participants with missing responses on age, 3314 LBC were identified and retained for final analyses. The definition of left-behind status was consistent with previous studies on Chinese adolescents [5,13,14,15]. The study was approved by participating schools and the Human Research Ethics Committee of the leading authors’ University.

### 2.2. Measures

We collected participants’ socio-demographic information, eating habits, and mental health problems.

- Socio-demographic information: sex, age, grade (Grade 1, Grade 2, or Grade 3), residence (urban or rural), sibling status (the only child or not), parental status, parental education, family structure, and family income.
- Eating habits: eating habits were collected by five items from the nutritional subscale of the Chinese version of the Health Promoting Lifestyle Profile-II (HPLP-II) [16]. Specifically, these items asked about the frequency of eating cereals, fruit, vegetables, protein, and breakfast. All items were scored according to a 4-point Likert scale (1 = never, 2 = sometimes; 3 = often; 4 = always).
- Mental health problems: the severity of insomnia, depression, and anxiety were adopted as primary outcome variables in this study. The severity of insomnia was measured by the Chinese version of the Youth Self-Rating Insomnia Scale [17]. The YSIS consisted of 8 items with each item that is rated on a 5-point scale to form a possible total score ranging from 8 to 40. The recommended cutoffs for insomnia severity are below: <22 (normal), 22–25 (mild), 26–29 (moderate), ≤30 (severe) [17]. The severity of depression was measured by the Chinese version of the 9-item Patient Health Questionnaire-9 item (PHQ-9) [18]. Each item can earn 0 to 3 points (0 = not at all, 3 = nearly every day), and the total score of the PHQ-9 can range from 0 to 27. The recommended cutoffs for depression severity are below: <4 (normal), 5–9 (mild), 10–14 (moderate), 15–19 (moderately severe), 20–27 (severe) [19]. The severity of anxiety was measured by the Generalized Anxiety Disorder Scale-7 item (GAD-7) [20]. Each item can earn 0 to 3 points (0 = not at all, 3 = nearly every day), which gives the GAD-7 a possible range of 0 to 21. The recommended cutoffs for anxiety severity are below: <4 (normal), 5–9 (mild), 10–14 (moderate), 15–21 (severe) [21].

The HPLP-II, YSIS, PHQ-9, and GAD-7 have presented good reliability and validity in previous studies on Chinese adolescents [17,22,23,24].

### 2.3. Statistical Analyses

Analyses were stratified by sex. Descriptive results of socio-demographic information, eating habits, and mental health problems were presented as counts (*n*) and percentages (%). Sex differences in eating habits and mental health problems were examined using χ^2^. Partial correlation analysis was performed to preliminarily investigate the correlation between eating habits and mental health problems, adjusting for age, grade, sibling status, parental education, family structure, and family income. The associations between daily eating behaviors and mental health problems were analyzed using generalized linear models with Poisson distribution and log link, adjusted for the covariates same as the partial correlation analyses. Specifically, we established two models: model 1 included the frequency of eating carbohydrates, fruits, vegetables, and protein, and model 2 included the frequency of eating breakfast. Risk ratio (RR) and the 95% confidence interval (CI) were reported. Significance levels were set as *p* < 0.05. SPSS (version 26) was used for data analyses.

## 3. Results

Among 3314 LBC included, there were 1455 (43.9%) males and 1859 (56.1%) females. They were aged 12–17 years, and the mean age was 14.21 (SD = 0.96) in males and 14.14 (SD = 0.94) in females, respectively. Detailed information about sample characteristics is provided in Table 1.

Table 2 describes eating habits and mental health problems. Most participants chose “sometimes” on all items of eating habits. The proportion of participants reported “routinely” on eating habits studied was low (males: 12.2% (fruits) to 24.3% (breakfast), females: 8.7% (fruits) to 19.7% (carbohydrates)). The prevalence of mental health outcomes with moderate severity and above was 28.4% (insomnia), 28.1% (depression), and 17.1% (anxiety), respectively. Compared to males, a lower proportion of reporting “routinely” on eating items was observed in females (all *p* < 0.01). In contrast, the prevalence of mental health problems with a moderate or above severity was higher in females (all *p* < 0.001).

Table 3 presents the results of the preliminary analysis of the association between eating habits and mental health problems. After adjusting for covariates, the frequency of eating carbohydrates did not present a significant correlation with mental health problems. The frequency of eating fruits was significantly inversely correlated to the severity of insomnia in both sexes, depression in females, and anxiety in females. The frequencies of eating vegetables and protein were significantly inversely correlated to the severity of insomnia, depression, and anxiety in females. In addition, the frequency of eating breakfast presented a significant correlation with the severity of insomnia in both sexes, depression in both sexes, and anxiety in females.

Table 4 shows the results of the regression model for insomnia severity. The “routinely” frequency was the reference. In males, sometimes eating breakfast was associated with a significantly higher risk of insomnia severity (RR = 1.20, 95% CI = 1.08–1.34, *p* = 0.001). In females, never and sometimes eating fruit (never: RR = 1.36, 95% CI = 1.13–1.65, *p* = 0.001; sometimes: RR = 1.17, 95% CI = 1.00–1.36, *p* = 0.046), and never and sometimes eating breakfast (never: RR = 1.43, 95% CI = 1.24–1.65, *p* < 0.001; sometimes: RR = 1.30, 95% CI = 1.18–1.43, *p* < 0.001) were associated with a significantly higher risk of insomnia severity.

Table 5 shows the results of the regression model for depression severity. The “routinely” frequency was the reference. In males, sometimes eating breakfast was associated with a significantly higher risk of depression severity (RR = 1.17, 95% CI = 1.06–1.30, *p* = 0.003). In females, never and sometimes eating fruit (never: RR = 1.34, 95% CI = 1.12–1.62, *p* = 0.002; sometimes: RR = 1.18, 95% CI = 1.01–1.37, *p* = 0.033), and never and sometimes eating breakfast (never: RR = 1.38, 95% CI = 1.19–1.59, *p* < 0.001; sometimes: RR = 1.29, 95% CI = 1.18–1.42, *p* < 0.001) were associated with a significantly higher risk of depression severity.

Table 6 shows the results of the regression model for anxiety severity. In males, no significant associations were observed. In females, compared to the “routinely” frequency, never eating fruit (RR = 1.23, 95% CI = 1.00–1.50, *p* = 0.050), and never and sometimes eating breakfast (never: RR = 1.30, 95% CI = 1.11–1.52, *p* = 0.001; sometimes: RR = 1.21, 95% CI = 1.09–1.33, *p* *<* 0.001) were associated with a significantly higher risk of anxiety severity.

## 4. Discussion

Using a sample of Chinese LBC, the current study aimed to investigate the situation of eating habits and their association with mental health problems, including the severity of depression, anxiety, and insomnia. To our knowledge, this is one of the first studies to examine the roles of eating habits and mental health status in Chinese LBC across the literature. The results mainly indicated that eating habits in Chinese LBC were not optimal, with a low proportion of routinely adopting healthy eating habits in this study. Eating breakfast and fruit was associated with significantly lower risks of mental health problems. The associations were more pronounced in females than males. Further discussion is presented below in detail.

We measured the frequency of eating carbohydrates, fruits, vegetables, protein, and breakfast. All of these are necessities in daily life, but the proportion of reporting a frequency being “routinely” was low. As the participants were LBC living in economically disadvantageous areas of China, the current results support previous findings that adolescents in a low socioeconomic position were more likely to have poor diet quality [25,26]. In addition, an even lower proportion of reporting higher frequency was observed in females compared to males. This may be because female adolescents were more likely to pay attention to their body image and tend to reduce their food intake to maintain the ideal figure or lose weight [27]. Further, the mental health problems were more severe in females than males, which highlights the need to give more psychological care to female LBC.

Eating breakfast was a significant factor for lower risks of mental health problems in LBC. In our study, compared with LBC who routinely have breakfast, those with a lower frequency (e.g., sometimes eating breakfast) had higher risks for insomnia and depression severity in both sexes, as well as anxiety severity in females. This suggests that routinely eating breakfast would be a protective factor against mental health problems in Chinese LBC. This research finding is consistent with similar studies not focusing on LBC [28,29,30]. Recently, a meta-analysis has concluded that skipping breakfast in adolescents was positively associated with higher risks of depression symptoms and anxiety [10]. There is also evidence that eating breakfast was significantly associated with insomnia in adolescents [31,32]. Skipping breakfast may be a result of poor sleep at night and late-wake time [33], causing lack of energy and further leading to tiredness in the daytime. Although there is no consensus on the mechanisms that explain why skipping breakfast might harm mental health, a large number of studies have shown that skipping breakfast could increase the risk of obesity, which often occurs with mental health problems and may further increase the risk of mental health problems [34,35]. In addition, we noted that the association between the frequency of breakfast and mental health problems was more pronounced in females. This is an interesting research finding. Nevertheless, previous studies lacked sex-stratified evidence [10], which limits our understanding of sex differences in the association between eating breakfast and mental health problems in LBC. Future studies are encouraged to address the mechanism underlying eating breakfast and mental health problems in LBC, especially considering sex differences.

Additionally, the current study found that eating fruit routinely was associated with lower risks of mental health problems, but this was only found in girls. This is partly supported by previous evidence that fruit intake was significantly associated with lower risks of depression and anxiety [8,9]. No significant associations between other eating habits (i.e., carbohydrates, vegetables, and protein) and mental health problems after adjusting for covariates. This may be accounted for by the fact that fruit intake occurred at different times from other foods (carbohydrates, vegetables, and protein), and generally not within a composite meal. Therefore, the frequency of fruit intake may be a key marker of a healthy dietary pattern. The nutritional components in fruits (e.g., vitamin) could dampen some of the detrimental effects of stress on mental health [36]. In addition, similar to the above finding of eating breakfast, the association between eating fruit and mental health was stronger in females. This is in line with the evidence of a multi-national study that the associations between vegetable intake and depression/anxiety were significant among females but not among males [9]. Nevertheless, the sex difference in the association needs more research analyzing the association between fruit intake and mental health for a clear explanation.

### Strengths, Limitations, and Future Directions

Some strengths of this study should not be neglected. One was the large sample size (more than 3000), which is helpful to interpret our research findings. The second was that our study focused on the association between eating habits and mental health in LBC, advancing knowledge in the relevant research fields and providing evidence to policymakers. In addition, we measured the fruit intake and vegetable intake individually which can provide more specific insights into the eating habits of LBC compared to previous research examining both food groups together. It is important to examine the consumption of fruit and vegetable separately, not only because fruit and vegetables can provide different nutrients and benefits, but also because adolescents often have different preferences and practices of eating fruit and vegetables [37]. Different roles of fruit and vegetable intake observed in the current study also provide a reason to examine the two food groups individually in future studies.

This study is not without limitations. First of all, the cross-sectional design in this study precluded drawing any causal inference on the association between the independent variables and the outcomes. More studies using an advanced design, such as longitudinal and intervention studies, are needed to fully investigate the effects of dietary habits on mental health or the direction of the association between dietary habits and mental health. Second, considering the large sample size involved, a brief assessment of diet habits was adopted in this study. The non-significant correlation (Table 3) between the intake of carbohydrates and mental health problems may partly be explained by the simple assessment. The specific type of dietary carbohydrates might have different effects on mental health, owing to various nutrients contained in different types of carbohydrates. For example, carbohydrates with different glycemic indexes might have different relations with mental health [38]. More accurate and comprehensive dietary assessment tools, such as 24-h dietary recalls and semi-quantitative food frequency questionnaires [39,40], are recommended in future studies to obtain more nuanced information. Third, our study was based on adolescents only from Guangxi Province, which, in turn, makes generalizability of the research findings limited. We expect that studies on this topic can be carried out in different regions (especially those with a high proportion of LBC) to verify the universality of the conclusions reached in this study. Fourth, owing to limited research conditions (e.g., the COVID-19) and resources, we failed to use random sampling to determine the sample in our study. Future research would benefit from using a random sampling approach and recruiting participants from multiple regions. Fifth, although we have included socio-demographic variables as covariates in the current study, other potential correlates (e.g., the mental health status of the caregiver) that may impact adolescents’ mental health were not considered in this study. Subsequent research can be more comprehensive by collecting more information on covariates to improve the power of statistical analysis. Additionally, our data were collected in May 2020, although the COVID-19 pandemic status was generally under control in China at that time, the diet habits may be affected by the pandemic and we did not measure the diet habits before the unexpected pandemic. Future studies are urged to address these study limitations and then provide more meaningful evidence for policy development and implementation for the healthy growth of Chinese LBC.

## 5. Conclusions

Generally, the dietary quality was undesirable in Chinese LBC. The frequency of fruit intake and breakfast consumption were two important dietary factors of the mental health status of LBC. The associations between eating habits and mental health problems were particularly stronger in females rather than males. Considering that females reported poorer diet patterns than males in this study, to reduce the occurrence of mental health problems in female LBC, improving diet quality (particularly avoiding skipping breakfast and improving fruit intake) would be a particularly effective approach.

## Figures and Tables

**Table 1:** Socio-demographic information of the sample.

Variables	Males (N = 1455)		Females (N = 1859)	
	*n*	%	*n*	%
Grade				
Grade 1	623	42.8	741	39.9
Grade 2	469	32.2	617	33.2
Grade 3	363	24.9	501	26.9
Residence				
Urban	203	14.0	204	11.0
Rural	1252	86.0	1655	89.0
Sibling status				
Only child	343	23.6	281	15.1
Non-only child	1112	76.4	1578	84.9
Family structure				
Full	1136	78.1	1460	78.5
Divorced	206	14.2	271	14.6
Other	113	7.8	128	6.9
Paternal education				
Junior middle school or below	1145	78.7	1566	84.2
High school or equivalent	228	15.7	234	12.6
Bachelor or equivalent	52	3.6	42	2.3
Master or above	30	2.1	17	0.9
Maternal education				
Junior middle school or below	1164	80.0	1603	86.2
High school or equivalent	206	14.2	206	11.1
Bachelor or equivalent	44	3.0	33	1.8
Master or above	41	2.8	17	0.9
Family per capita monthly income (yuan)				
Less than 1000	184	12.6	239	12.9
1000–1999	237	16.3	424	22.8
2000–2999	298	20.5	381	20.5
3000–3999	323	22.2	414	22.3
4000–4999	136	9.3	163	8.8
5000–5999	79	5.4	91	4.9
More than 6000	198	13.6	147	7.9

**Table 2:** Sex difference of eating habits and mental health problems.

Variables	Males		Females			Variables	Males		Females		
	*n*	%	*n*	%	*p*		*n*	%	*n*	%	*p*
Carbohydrates						Insomnia severity					
Never	167	11.5	154	8.3	0.001	Normal	993	68.2	1011	54.4	<0.001
Sometimes	534	36.7	783	42.1		Mild	196	13.5	321	17.3	
Often	431	29.6	555	29.9		Moderate	135	9.3	258	13.9	
Routinely	323	22.2	367	19.7		Severe	131	9.0	269	14.5	
Fruits						Depression severity					
Never	201	13.8	203	10.9	<0.001	Normal	798	54.8	797	42.9	<0.001
Sometimes	744	51.1	1106	59.5		Mild	407	28.0	539	29.0	
Often	333	22.9	388	20.9		Moderate	130	8.9	284	15.3	
Routinely	177	12.2	162	8.7		Moderately severe	85	5.8	160	8.6	
Vegetables						Severe	35	2.4	79	4.2	
Never	137	9.4	120	6.5	0.002	Anxiety severity					
Sometimes	530	36.4	762	41.0		Normal	984	67.6	1044	56.2	<0.001
Often	503	34.6	640	34.4		Mild	322	22.1	498	26.8	
Routinely	285	19.6	337	18.1		Moderate	93	6.4	204	11.0	
Protein						Severe	56	3.8	113	6.1	
Never	141	9.7	116	6.2	<0.001						
Sometimes	588	40.4	1023	55.0							
Often	473	32.5	501	26.9							
Routinely	253	17.4	219	11.8							
Breakfast											
Never	156	10.7	128	6.9	<0.001						
Sometimes	538	37.0	919	49.4							
Often	407	28.0	472	25.4							
Routinely	354	24.3	340	18.3							

**Table 3:** Partial correlations between eating habits and mental health problems.

Variables	Insomnia Severity		Depression Severity		Anxiety Severity	
	Males	Females	Males	Females	Males	Females
Carbohydrates	0.03	−0.01	−0.02	−0.03	0.02	−0.02
Fruits	−0.05 *	−0.14 ***	−0.04	−0.15 ***	−0.03	−0.10 ***
Vegetables	−0.01	−0.07 **	−0.03	−0.11 ***	−0.01	−0.08 **
Protein	−0.01	−0.07 **	−0.03	−0.08 **	0.00	−0.06 *
Breakfast	−0.09 **	−0.19 ***	−0.07 **	−0.19 ***	−0.03	−0.15 ***

Adjusted for age, grade, sibling status, parental education, family structure, and family income. * *p* < 0.05, ** *p* < 0.01, *** *p* < 0.001.

**Table 4:** Regression model of the association between eating habits and insomnia severity.

Variables	Males				Females			
	RR	95% CI		*p*	RR	95% CI		*p*
		LL	UL			LL	UL	
Carbohydrates ^1^								
Never	0.87	0.68	1.10	0.234	0.85	0.72	1.00	0.054
Sometimes	0.89	0.77	1.04	0.130	0.95	0.85	1.06	0.360
Often	0.95	0.82	1.10	0.462	0.96	0.85	1.07	0.424
Fruits ^1^								
Never	1.25	0.99	1.57	0.058	**1.36**	**1.13**	**1.65**	**0.001**
Sometimes	1.08	0.90	1.29	0.402	**1.17**	**1.00**	**1.36**	**0.046**
Often	1.01	0.83	1.22	0.934	1.00	0.84	1.18	0.959
Vegetables ^1^								
Never	0.90	0.67	1.21	0.474	1.04	0.86	1.27	0.673
Sometimes	1.02	0.86	1.20	0.852	0.98	0.86	1.11	0.733
Often	0.90	0.76	1.06	0.203	0.97	0.86	1.10	0.641
Protein ^1^								
Never	0.99	0.76	1.29	0.954	1.02	0.82	1.27	0.844
Sometimes	1.09	0.93	1.28	0.312	1.10	0.95	1.27	0.200
Often	1.09	0.93	1.28	0.302	1.06	0.91	1.23	0.456
Breakfast ^2^								
Never	1.07	0.92	1.25	0.372	**1.43**	**1.24**	**1.65**	**<0.001**
Sometimes	**1.20**	**1.08**	**1.34**	**0.001**	**1.30**	**1.18**	**1.43**	**<0.001**
Often	1.04	0.92	1.17	0.514	1.05	0.94	1.18	0.354

RR: risk ratio, LL: lower limit, UL: upper limit. ^1^ Model 1 included age, grade, sibling status, parental education, family structure, family income, and the frequency of eating carbohydrates, fruit, vegetables, and protein. ^2^ Model 2 included age, grade, sibling status, parental education, family structure, family income, and the frequency of eating breakfast. The reference category is “routinely” frequency. Significant *p* values and corresponding RR and 95% CI are bold.

**Table 5:** Regression model of the association between eating habits and depression severity.

Variables	Males				Females			
	RR	95% CI		*p*	RR	95% CI		*p*
		LL	UL			LL	UL	
Carbohydrates ^1^								
Never	1.02	0.81	1.27	0.886	0.92	0.79	1.08	0.314
Sometimes	0.98	0.85	1.14	0.817	0.95	0.85	1.06	0.372
Often	1.02	0.88	1.18	0.792	0.98	0.88	1.10	0.735
Fruits ^1^								
Never	1.18	0.94	1.47	0.147	**1.34**	**1.12**	**1.62**	**0.002**
Sometimes	1.00	0.84	1.19	0.984	**1.18**	**1.01**	**1.37**	**0.033**
Often	1.00	0.83	1.20	0.974	1.07	0.91	1.26	0.426
Vegetables ^1^								
Never	0.85	0.64	1.13	0.256	1.09	0.89	1.32	0.403
Sometimes	1.01	0.86	1.18	0.954	1.11	0.98	1.26	0.101
Often	0.89	0.76	1.05	0.156	1.03	0.92	1.16	0.598
Protein ^1^								
Never	0.98	0.76	1.26	0.886	0.95	0.77	1.17	0.63
Sometimes	1.13	0.96	1.32	0.133	1.00	0.87	1.15	0.957
Often	1.09	0.93	1.28	0.274	0.95	0.83	1.10	0.497
Breakfast ^2^								
Never	1.06	0.92	1.24	0.422	**1.38**	**1.19**	**1.59**	**<0.001**
Sometimes	**1.17**	**1.06**	**1.30**	**0.003**	**1.29**	**1.18**	**1.42**	**<0.001**
Often	1.07	0.96	1.20	0.226	1.08	0.97	1.20	0.160

RR: risk ratio, LL: lower limit, UL: upper limit. ^1^ Model 1 included age, grade, sibling status, parental education, family structure, family income, and the frequency of eating carbohydrates, fruit, vegetables, and protein. ^2^ Model 2 included age, grade, sibling status, parental education, family structure, family income, and the frequency of eating breakfast. The reference category is “routinely” frequency. Significant *p* values and corresponding RR and 95% CI are bold.

**Table 6:** Regression model of the association between eating habits and anxiety severity.

Variables	Males				Females			
	RR	95% CI		*p*	RR	95% CI		*p*
		LL	UL			LL	UL	
Carbohydrates ^1^								
Never	0.85	0.66	1.10	0.223	0.94	0.79	1.11	0.456
Sometimes	0.96	0.82	1.12	0.582	0.94	0.83	1.06	0.300
Often	0.98	0.84	1.15	0.815	0.94	0.83	1.06	0.311
Fruits ^1^								
Never	1.15	0.90	1.46	0.266	**1.23**	**1.00**	**1.50**	**0.050**
Sometimes	1.07	0.89	1.29	0.479	1.11	0.95	1.31	0.194
Often	1.03	0.84	1.26	0.798	1.06	0.89	1.26	0.549
Vegetables ^1^								
Never	0.97	0.71	1.32	0.829	1.03	0.83	1.28	0.771
Sometimes	1.00	0.84	1.18	0.964	1.07	0.94	1.23	0.314
Often	0.91	0.77	1.08	0.297	0.99	0.87	1.13	0.875
Protein ^1^								
Never	1.05	0.80	1.38	0.744	0.99	0.78	1.25	0.914
Sometimes	1.05	0.89	1.25	0.557	1.03	0.88	1.20	0.695
Often	1.12	0.95	1.33	0.183	1.02	0.87	1.19	0.806
Breakfast ^2^								
Never	0.99	0.84	1.17	0.910	**1.30**	**1.11**	**1.52**	**0.001**
Sometimes	1.10	0.98	1.23	0.115	**1.21**	**1.09**	**1.33**	**<0.001**
Often	1.05	0.93	1.18	0.430	1.06	0.95	1.19	0.287

RR: risk ratio, LL: lower limit, UL: upper limit. ^1^ Model 1 included age, grade, sibling status, parental education, family structure, family income, and the frequency of eating carbohydrates, fruit, vegetables, and protein. ^2^ Model 2 included age, grade, sibling status, parental education, family structure, family income, and the frequency of eating breakfast. The reference category is “routinely” frequency. Significant *p* values and corresponding RR and 95% CI are bold.

## Data Availability

The data analyzed in the current study are available from the corresponding author on reasonable request.
